# The Dual Roles of MAGE-C2 in p53 Ubiquitination and Cell Proliferation Through E3 Ligases MDM2 and TRIM28

**DOI:** 10.3389/fcell.2022.922675

**Published:** 2022-07-19

**Authors:** Yunshan Liu, Beibei Cao, Liqiao Hu, Jingjing Ye, Wei Tian, Xiaojing He

**Affiliations:** Key Laboratory of Molecular Biophysics of the Ministry of Education, College of Life Science and Technology, Huazhong University of Science and Technology, Wuhan, China

**Keywords:** cell proliferation, p53 ubiquitination, MDM2, TRIM28, MAGE-C2

## Abstract

The tumor suppressor p53 is critical for the maintenance of genome stability and protection against tumor malignant transformation, and its homeostasis is usually regulated by ubiquitination. MDM2 is a major E3 ligase of p53 ubiquitination, and its activity is enhanced by TRIM28. TRIM28 also independently ubiquitinates p53 as an E3 ligase activated by MAGE-C2. Moreover, MAGE-C2 is highly expressed in various cancers, but the detailed mechanisms of MAGE-C2 involved in MDM2/TRIM28-mediated p53 ubiquitination remain unknown. Here, we found that MAGE-C2 directly interacts with MDM2 through its conserved MHD domain to inhibit the activity of MDM2 on p53 ubiquitination. Furthermore, TRIM28 acts as an MAGE-C2 binding partner and directly competes with MAGE-C2 for MDM2 interaction, thus releasing the inhibitory role of MAGE-C2 and promoting p53 ubiquitination. MAGE-C2 suppresses cell proliferation in TRIM28-deficient cells, but the overexpression of TRIM28 antagonizes the inhibitory role of MAGE-C2 and accumulates p53 ubiquitination to promote cell proliferation. This study clarified the molecular link of MAGE-C2 in two major E3 systems MDM2 and TRIM28 on p53 ubiquitination. Our results revealed the molecular function of how MAGE-C2 and TRIM28 contribute to p53 ubiquitination and cell proliferation, in which MAGE-C2 acts as a potential inhibitor of MDM2 and TRIM28 is a vital regulator for MAGE-C2 function in p53 protein level and cell proliferation. This work would be helpful to understand the regulation mechanism of tumor suppressor p53.

## Introduction

As a well-known tumor suppressor, p53 protein is important for guarding genome stability and preventing against tumor malignant transformation (Kulaberoglu et al., 2016; Ou and Schumacher, 2018). In response to cellular stress or DNA damage, p53 functions as a transcription factor and activates a multitude of target genes, such as *p21* and *BAX*, to induce apoptosis and cell cycle arrest (Meek, 2004). p53 is inactivated in various tumors by causing mutations in the *TP53* locus, and it is regulated by multiple viral and cellular proteins (Gritsenko et al., 2017; Sullivan et al., 2018). The mouse double minute 2 (*MDM2*) gene has been identified in various cancers and is known as a major regulator of p53 (Oliner et al., 2016; Hou et al., 2019). Extensive studies have established that MDM2 acts as an onco-protein and inhibits p53 activity through two distinct mechanisms. MDM2 interacts with p53 and blocks the transcriptional activity of p53 for target genes (Haupt et al., 1997). MDM2 also functions as a RING domain E3 ligase to mediate the ubiquitination of p53 (Kubbutat et al., 1997; Zhao et al., 2015). The multidomain protein TRIM28 enhances MDM2 activity and further facilitates the ubiquitination and degradation of p53 (Wang et al., 2005). Interestingly, other reports also suggested that TRIM28 acts as an independent E3 ligase to mediate p53 ubiquitination, in which MAGE-C2, a member of melanoma-associated antigens (MAGE) family, is required to activate the ligase function of TRIM28 (Doyle et al., 2010).

As cancer-associated antigens, MAGE family has been found to promote cell proliferation and tumorigenesis in multiple cancers (Xiao, 2004; Sang et al., 2011; Florke Gee et al., 2020). MAGE family consists of more than 60 members in *Homo sapiens*, which are divided into type I and type II classes based on expression pattern and share a conserved MAGE homology domain (MHD) (Lucas et al., 1998). However, different members of MAGE family play diverse functions. For example, MAGE-A2 inhibits p53 ubiquitination and induces cell death (Marcar et al., 2015), and MAGE-D1 upregulates p53 and inhibits the proliferation and migration of breast cancer cells (Du et al., 2009). Whereas the aberrant expression of MAGE-C2 in cancers is positively correlated with tumorigenesis progress and p53 ubiquitination (Xiao, 2004; Doyle et al., 2010; Sang et al., 2011; Florke Gee et al., 2020). In addition to facilitate p53 ubiquitination (Doyle et al., 2010), MAGE-C2 also promotes fructose 1,6-bisphosphate (FBP1) ubiquitination by TRIM28 and results in Warburg effects and HCC progression (Jin et al., 2017). However, whether MAGE-C2 is involved in the MDM2-mediated p53 ubiquitination and performs different functions with distinct E3 ligases remain unknown.

In this study, we found that MAGE-C2 directly interacts with MDM2 and inhibits its ligase activity through the conserved SBC site on MHD domain. But MAGE-C2 fails to maintain p53 protein level in HeLa cells and actually increases cell proliferation. We then unveiled the reason that TRIM28 competitively breaks the interaction between MAGE-C2 and MDM2 and antagonizes the inhibitory effect of MAGE-C2 on MDM2-dependent p53 ubiquitination. Moreover, we analyzed the cellular function of TRIM28 and MAGE-C2 in different types of cells and found that the overexpression of MAGE-C2 accumulates p53 protein level and decreases cell proliferation in TRIM28-depleted cells. However, the exogenous TRIM28 significantly downregulates p53 protein level and promotes cancer cell proliferation. Collectively, our results proposed a relationship between TRIM28 and MDM2 on p53 ubiquitination that TRIM28 preferentially binds to MAGE-C2, prevents the inhibition on MDM2, and ultimately accelerates the ubiquitination of p53. This study also suggested divergent roles of MAGE-C2 in the regulation of E3 ligases TRIM28 and MDM2 during cell proliferation and tumorigenesis.

## Materials and Methods

### Protein Expression and Purification

Synthetic genes encoding MAGE-C2 FL (residues 1–373), MAGE-C2 MHD (residues 140–350), MAGE-C2 mutants, and p53 (residues 1–393) were cloned into pGEX-6p-1 plasmid (Novagen), with N-terminal glutathione S-transferase (GST)-tag followed by a 3C protease cleavage site. The full lengths of MDM2 (residues 1–491), UbcH2 (residues 1–183), and UbcH5B (residues 1–147) were cloned into SUMO vector (modified pET-28a).

Ubiquitin was subcloned into pET-29a vector (Invitrogen). The *E. coli* BL21 (DE3) cells were transformed with the respective expression construct. Starter cultures were incubated at 37°C to an optical density (OD 600) of 0.6–0.8 in terrific broth (TB) medium, and the incubator temperature was lowered to 16°C. Protein expression was induced with 0.2 mM isopropyl-β-D-thiogalactopyranoside (IPTG). After 16 h, cells were harvested using centrifugation and stored at −80°C. All subsequent steps were performed at 4°C.

To purify the various MAGE-C2 truncations, cells were resuspended in lysis buffer containing 20 mM Tris, pH 8.0, 300 mM NaCl, 0.2 mM TCEP, and 1 mM EDTA. The cells were lysed by using sonication and clarified using centrifugation (40 min, 18,000 rpm). The supernatant was applied to a glutathione Sepharose column (GE Healthcare) equilibrated in wash buffer (20 mM Tris, pH 8, 300 mM NaCl, and 0.2 mM TCEP) for one hour at rotator. Then the column was washed with 30 column volumes (CVs) of wash buffer and then exchanged to cut buffer (20 mM Tris, pH 8, 150 mM NaCl, and 0.2 mM TCEP). GST tag was removed by incubating the 3C protease overnight, and the cleaved proteins were collected. The sample was purified by using size-exclusion chromatography with a Superdex (10/300) column (GE Healthcare) pre-equilibrated in 20 mM Tris, pH 8, 150 mM NaCl, and 0.2 mM TCEP. SUMO-MDM2 was expressed and purified as GST proteins except using HisTrap nickel-affinity column (GE Healthcare) in affinity chromatography step. Both lysis buffer and wash buffer are with additional 25 mM imidazole, and elution buffer with 250 mM imidazole. SUMO-MDM2 fusion protein was eluted directly without tag cleavage by protease on the column.

All protein samples were then subjected to Superdex 200 (10/300) column (GE Healthcare) pre-equilibrated in 20 mM Tris, pH 8, 150 mM NaCl, and 0.2 mM TCEP with a flow rate of 0.3 ml/min at 4°C for final purification.

### Glutathione S-Transferase Pull-Down Assay

Pull-down assays were performed by incubating 20 μg of purified GST-tagged MAGE-C2 truncations with glutathione Sepharose beads (GE Healthcare) for one hour in binding buffer (20 mM Tris, pH 8, 150 mM NaCl, 0.2 mM TCEP, and 0.02% Triton-100). Bound beads were blocked for one hour in binding buffer containing 5% milk powder. *In vitro* translated proteins (5 μg) were then incubated with the bound beads for one hour in binding buffer containing 5% milk powder at 4°C. After three washes in binding buffer, the proteins were eluted in SDS sample buffer, boiled, subjected to SDS-PAGE, and detected using Coomassie staining or blotted with anti-MDM2. For quantitation of pull-down assays, films were scanned and subjected to densitometry analysis. Background binding to GST was subtracted from the binding to the relevant GST-tagged proteins and expressed as a percentage of the input protein.

### Western Blotting

After resolving the proteins with sodium dodecyl sulfate-polyacrylamide gel electrophoresis (SDS-PAGE), the proteins were transferred onto polyvinylidene difluoride membranes. Blots were blocked with 5% nonfat milk powder in TBST buffer for one hour at room temperature and incubated with primary antibody overnight at 4°C. Rabbit anti-p53 antibody or rabbit anti-MDM2 antibody was diluted 1:2000. Blots were incubated with fluorescent secondary antibodies for one hour at room temperature. Secondary antirabbit antibodies were diluted 1:3000. Blots were imaged by using an Odyssey CLx gel scanner (LI-COR Biosciences).

### *In Vitro* Ubiquitination Assay

*In vitro* ubiquitination assays were performed using 100 nM His-Ube1, 2.5 μM E2 (His-UbcH5B), 2.5 μM E3 (MDM2 or SUMO-MDM2), 5 μM MAGE-C2 (full length, truncation or mutants), 2.5 μM His-ubiquitin, 0.5 mM Mg-ATP, and 1 mM DTT in 1 × ubiquitination buffer (20 mM Tris, pH 8, and 150 mM NaCl). For those reactions examining substrate ubiquitination, 1 μM GST-p53 was added. Negative control reactions (–Mg-ATP) were set up as abovementioned except Mg-ATP was replaced with 5 mM EDTA. Reactions were stopped by the addition of 2 × SDS sample buffer after incubation at 37°C and subjected to SDS-PAGE and immunoblotting for ubiquitin chain formation, substrate ubiquitination (anti-p53), or E3 autoubiquitination (anti-MDM2).

### Cell Lines, Transfections, and Plasmids

HEK293T, HeLa, A549, and A375 cells were grown in DMEM (Invitrogen), H1299 cells were grown in 1,640 (Corning) supplemented with 10% fetal bovine serum (Gibco), 100 units/ml penicillin (Invitrogen), and 100 μg/ml streptomycin (Invitrogen). Plasmid transfection was performed with Lipofectamine 2000 (Invitrogen) reagents according to the manufacturer’s protocol. Plasmids used for the expression of human cDNAs were as follows: 0.5 μg wild type p53 in p3×FLAG-Myc-CMV-24, 1.5 μg HA-tagged ubiquitin in pCDNA3, 2 μg human wild type MDM2 in PIP-GFP, 4 μg Myc-tagged (full length, MHD domain and mutants) human MAGE-C2 in pCS2-Myc-Prc1-m, and 2 μg Myc-tagged human TRIM28 in pCS2-Myc-Prc1-m.

### Gene Silencing Using siRNA

TRIM28 expression was silenced using previously published siRNA oligo-nucleotides. TRIM28 siRNA sequence is as follows: 5′-GCAUGAACCCCUUGUGCUGdTdT-3′. siRNAs transfection was performed using Lipofectamine 2000 (Invitrogen) reagents according to the manufacturer’s protocol, and siTRIM28 oligonucleotides were purchased from GenePharma.

### Ubiquitination Assay in Cell

HEK293T cells were treated with 10 μM MG132 for eight hours before harvesting. Cells were lysed by ultrasonication in buffer (1 × phosphate-buffered saline (PBS), 1% Triton X-100, 1 mM EDTA, and 1 mM Tris (2-carboxyethyl) phosphine. Supernatant of cell lysates was incubated with anti-Flag M2-agarose redbeads (Sigma-Aldrich) and subjected to immunoprecipitation with HA antibody. The ubiquitination of p53 was detected using Western blot.

### Cell Proliferation Assay

HeLa cells were seeded into 12-well plates with appropriate cell density. After transfection, CCK-8 (Biosharp) assays were performed at the indicated time. Ten microliters of CCK-8 reagent were added to each well and incubated for an additional one hour at 37°C. The optical density at 450 nm was read by using a microplate reader (BioTek).

### Wound Healing Assay

Cells were seeded into 12-well culture plates at appropriate density until growing to 90% confluence. A linear scratch was created in the monolayer of cells with a sterile 10 µL micropipette tip. The detached cells were rinsed using PBS (phosphate buffer saline) for three times and then incubated in medium containing 1% FBS. The cells migrating to the wounded region were observed at 24 h. Each experiment was independently repeated three times.

### Live/Dead Cell Staining Assay

Cells were seeded into 12-well culture plates at appropriate density. After transfection, the cells were cultured for 36 h. Thawed and diluted appropriate volume of live/dead cell staining solution stock with PBS to make the work staining solution. Cells were washed with PBS for three times, equal volume of working staining solution was added, and then incubated at room temperature in dark for 15 min. Cells were observed immediately under a fluorescence microscope. Live cells should stain fluorescing green, while dead cells should appear red.

### Quantitative Real-Time Polymerase Chain Reaction Assay

Six-well culture plates were used for RNA extraction according to the manufacturer’s instructions. The extracted total RNA was used as a template and added with the *EVO* M-MLV RT premix Kit (ACCURATE BIOLOGY, China) according to the instruction. Then the reverse transcription was performed to synthesize complementary DNA (cDNA). The synthesized cDNA was used as a template for real-time PCR (qPCR). ACTB was used as an internal reference, and the relative expression level of TRIM28 was calculated with 2^−△△CT^ value. The sequence of primers used is as follows: ACTB-forward: CTGGAACGG TGAAGGTGACA, ACTB-reverse: AAG​GGA​CTT​CCT​GTA​ACA​ATG​CA. TRIM28-forward: TGT​TTC​CAC​CTG​GAC​TGT​CA, TRIM28-reverse: CCA​GCA​GTA​CAC​GCT​CAC​AT. MDM2-forward: CAT​TGT​CCA​TGG​CAA​AAC​AG, MDM2-reverse: GGCAGGGCTTATTC CTTTTC.

### Quantification and Statistical Analysis

All the statistical data are presented as mean ± SD. The independent Student *t* test was used to compare the difference between two preselected groups. Value of *p* < 0.05 was taken as statistical significance.

For quantification of p53 intensity, ImageJ was used to obtain numeric intensities of experimental subjects under investigation in Figures 3B, 5A, and Supplementary Figure S2A. A mask was generated to mark p53 and actin on the basis of the projected image. After background subtraction, the intensities of p53 and actin signals within the mask were obtained in number.

## Results

### MAGE-C2 Inhibits MDM2-Mediated p53 Ubiquitination *In Vitro* and in Cells

**FIGURE 1:**
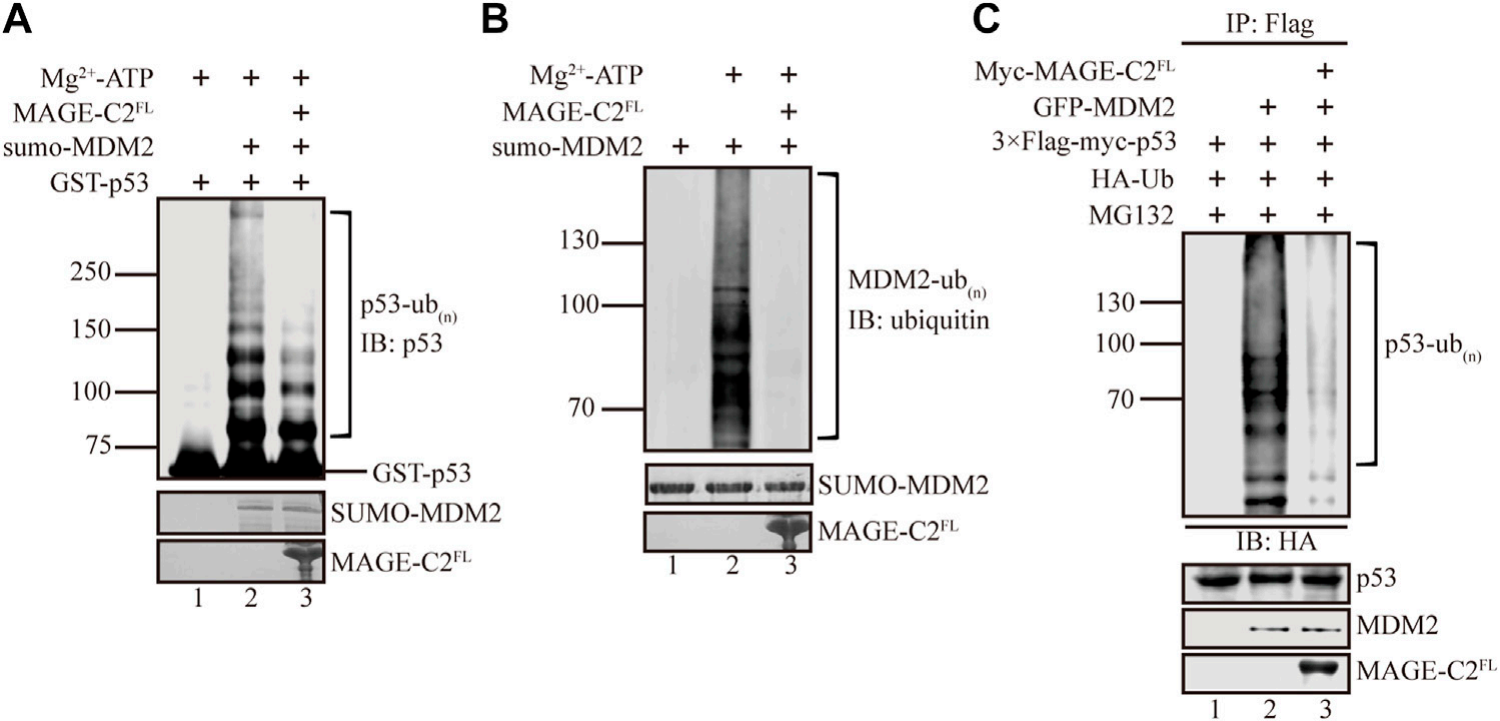
MAGE-C2 inhibits MDM2-mediated p53 ubiquitination *in vitro* and in cells. **(A)** *In vitro* ubiquitination assay. His-ubiquitin, ubiquitin E1, UbcH5B E2, Mg-ATP, GST-p53, and the indicated MAGE-C2 and MDM2 proteins were incubated for 40 min at 37°C and detected by using anti-p53 immunoblotting. **(B)** *In vitro* ubiquitination assay. His-ubiquitin, ubiquitin E1, UbcH5B E2, Mg-ATP, and the indicated MAGE-C2 and MDM2 proteins were incubated for 40 min at 37°C. His-ubiquitin was detected by using antiubiquitin immunoblotting. **(C)** Ubiquitination assay in cell. HEK293T cells were transfected with HA-ubiquitin and indicated plasmids. The level of p53 ubiquitination was determined by using anti-Flag M2-agarose redbeads purification and anti-HA Western blot.

MAGE-C2 has been shown to interact with TRIM28 and trigger its E3 ligase activity to downregulate the protein level of the tumor suppressor p53 (Doyle et al., 2010). Meanwhile, MDM2 acts as a major E3 ligase of p53 ubiquitination that promotes p53 degradation (Haupt et al., 1997; Wang et al., 2005; Oliner et al., 2016; Hou et al., 2019). This promoted us to be curious about whether MAGE-C2 participates in MDM2-mediated p53 ubiquitination. We first examined this based on *in vitro* ubiquitination assays. The sumo-tagged MDM2 and GST-tagged p53 were included as E3 ligase and substrate, respectively. In consistent with previous studies (Kubbutat et al., 1997), our results showed that MDM2 vigorously ubiquitinates p53 *in vitro,* which suggested high efficiency of the assay (Figure 1A, lane 2). The recombinant full length MAGE-C2 protein was introduced into the *in vitro* ubiquitination. Unexpectedly, MAGE-C2 significantly reduced the ubiquitination level of p53 (Figure 1A, lane 3), indicating that MAGE-C2 presumably inhibits p53 ubiquitination mediated by MDM2.

We wondered if MAGE-C2 might inhibit p53 ubiquitination by regulating the ligase activity of MDM2, and then *in vitro* auto-ubiquitination assay was performed. As the result showed, MDM2 exhibited dramatically auto-ubiquitination activity (Figure 1B, lane 2). In contrast, the addition of MAGE-C2 abolished the auto-ubiquitination activity of MDM2 (Figure 1B, lane 3). This observation suggested that MAGE-C2 potentially blocks the E3 ligase activity of MDM2 and sequentially inhibits p53 ubiquitination *in vitro*.

To further confirm the inhibitory role of MAGE-C2 in MDM2-mediated p53 ubiquitination, we performed a cell-based ubiquitination in HEK293T cells co-transfected with wild-type p53, ubiquitin, and MDM2. To feasibly detect the signals of p53 ubiquitination, the proteasome inhibitor MG132 was added to avoid the fast degradation of ubiquitinated p53. The results showed that MDM2 also efficiently ubiquitinated p53 in HEK293T cells (Figure 1C, lane 2). Moreover, in agreement with our *in vitro* results, the overexpression of exogenous MAGE-C2 significantly decreased the ubiquitination level of p53 (Figure 1C, lane 3), which confirmed that MAGE-C2 indeed inhibits MDM2-mediated p53 ubiquitination in cells. Thus, our findings demonstrated that MAGE-C2 functions as an inhibitor of MDM2 to restrain p53 ubiquitination.

### MAGE-C2 Directly Interacts With MDM2 and Inhibits Its Ligase Activity *via* the MHD Domain

**FIGURE 2:**
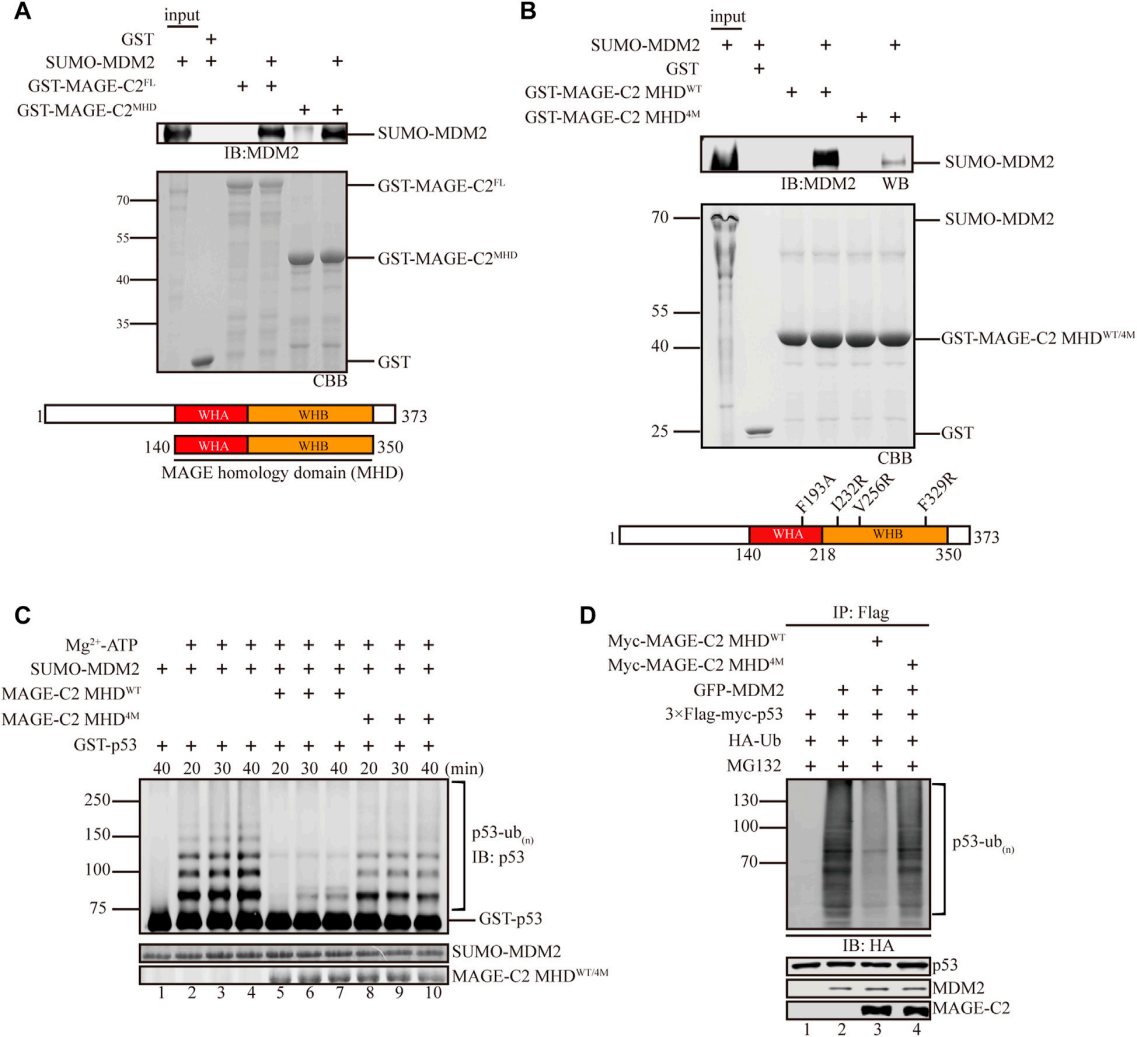
MAGE-C2 directly interacts with MDM2 and inhibits its ligase activity through the MHD domain. **(A)** Interactions between MAGE-C2 and MDM2 were validated by using GST pulldown assay. The indicated GST- and SUMO-tagged proteins were expressed in bacterium, and SUMO–MDM2 proteins were detected by using anti-MDM2 Western blot. GST was included as an irrelevant negative control protein. **(B)** Interactions between MAGE-C2 mutants and MDM2 were validated by using GST pulldown assay. The indicated GST-tagged MAGE-C2 proteins were incubated with *in vitro* translated SUMO–MDM2. Proteins were detected by using anti-MDM2 immunoblotting. **(C)** *In vitro* ubiquitination assay. His-ubiquitin, ubiquitin E1, UbcH5B E2, Mg-ATP, GST-p53, and the indicated MAGE-C2 and MDM2 proteins were incubated for specific time at 37°C and detected by using anti-p53 immunoblotting. **(D)** Ubiquitination assay in cells. HEK293T cells were transfected with HA-ubiquitin and indicated plasmids. The level of p53 ubiquitination was determined by using anti-Flag M2-agarose redbeads purification and anti-HA Western blot.

Next, we hypothesized that MAGE-C2 inhibits the ligase activity of MDM2 though direct interaction. To examine the connection between MAGE-C2 and MDM2, GST pull-down experiments were carried out using MAGE-C2 full length and MHD domain (residues 140–350), a conserved module usually mediated protein–protein interactions. As the results showed, both full length and MHD domain of MAGE-C2 directly bound to MDM2 with similar behaviors (Figure 2A), suggesting that MAGE-C2 indeed directly interacted with MDM2 mainly mediated by MHD domain. Consistent with the binding observation, both full length and MHD domain of MAGE-C2 exhibited the comparable inhibitory effect on the MDM2-dependent ubiquitination of p53 *in vitro* as well (Supplementary Figure S1A). Thus, our results indicated that MAGE-C2 directly interacts with MDM2 and inhibits its ligase activity through its MHD domain.

We then examined the binding sites between MAGE-C2 and MDM2 in more detail. According to the complex structure of MAGE-A11 and its substrate PCF11(PDB:6WJH) (Supplementary Figure S1B), a conserved substrate binding cleft (SBC) of MAGE-A11 has been shown to mediate substrate recognition and oncogenic activity (Yang et al., 2020). We introduced the corresponding SBC mutations into MAGE-C2 and created a mutant (F193A, I232R, V256R, and F329R, referred to MHD^4M^) of MAGE-C2 for the *in vitro* binding and ubiquitination experiments (Figure 2B). Indeed, MHD^4M^ dramatically diminished the binding ability to MDM2, in comparison with wild-type MAGE-C2 MHD (Figure 2B). As expected, MHD^4M^ almost lost the inhibition effect and restored the p53 ubiquitination mediated by MDM2 *in vitro* (Figure 2C). We also performed the cell-based ubiquitination assay in HEK293T cells to detect the effect of MHD^4M^. Consistent with the *in vitro* observation, MAGE-C2 MHD^WT^ robustly inhibited the p53 ubiquitination, whereas MHD^4M^ dramatically restored the ubiquitination level of p53 (Figure 2D). Taken together, the conserved substrate binding site (SBC) on MHD domain of MAGE-C2 is critical for the interaction with MDM2 and inhibition of MDM2-mediated p53 ubiquitination.

### The Inhibitory Role of MAGE-C2 in p53 Ubiquitination and Cell Proliferation Is Antagonized by TRIM28

MAGE-C2 has been shown to increase the cell proliferation and promote tumorigenesis (Chen et al., 2012; Curioni-Fontecedro et al., 2015; Zeng et al., 2017; Lamers et al., 2020). However, the molecular evidence here implied that MAGE-C2 inhibits the ubiquitination of the tumor suppressor p53 and presumably restrains tumor cell proliferation. We then assessed the function of MAGE-C2 in regulating p53 protein level and cell proliferation. The HeLa cell line was first chosen, since MAGE-C2 is spontaneously absent which may reduce the background effect. Surprisingly, the overexpression of MAGE-C2 in HeLa cells actually downregulated the protein level of p53 (Supplementary Figure S2A) and facilitated cell proliferation based on cell counting kit-8 (CCK-8) assay (Supplementary Figure S2B), which is quite contradictory with abovementioned *in vitro* evidence. The controversy between molecular evidence and functional phenotype led us to consider which regulators antagonize the inhibitory role of MAGE-C2 on E3 ligase MDM2 in HeLa cells.

It has been shown that TRIM28 not only associates with MAGE-C2 but also enhances the E3 ligase activity of MDM2 (Wang et al., 2005; Doyle et al., 2010), which implied that TRIM28 is possibly involved in blocking the tumor suppression of MGAE-C2. We first detected the complicated links among MAGE-C2, MDM2, and TRIM28. MAGE-C2 indeed directly interacted with TRIM28 (Supplementary Figure S2C) and activated TRIM28 to ubiquitinate p53 in cells (Supplementary Figure S2D). Moreover, TRIM28 definitely enhanced MDM2-dependent p53 ubiquitination in the *in vitro* assays only containing the specific E2 for MDM2 (Supplementary Figure S2E), and TRIM28 alone did not exhibit E3 ligase activity on p53 in MAGE-C2–negative cells (Supplementary Figure S2F). These findings confirmed the functions of TRIM28 in p53 ubiquitination (Wang et al., 2005; Doyle et al., 2010) and indicated the potential roles of TRIM28 to antagonize MAGE-C2 in MDM2-dependent p53 ubiquitination.

**FIGURE 3:**
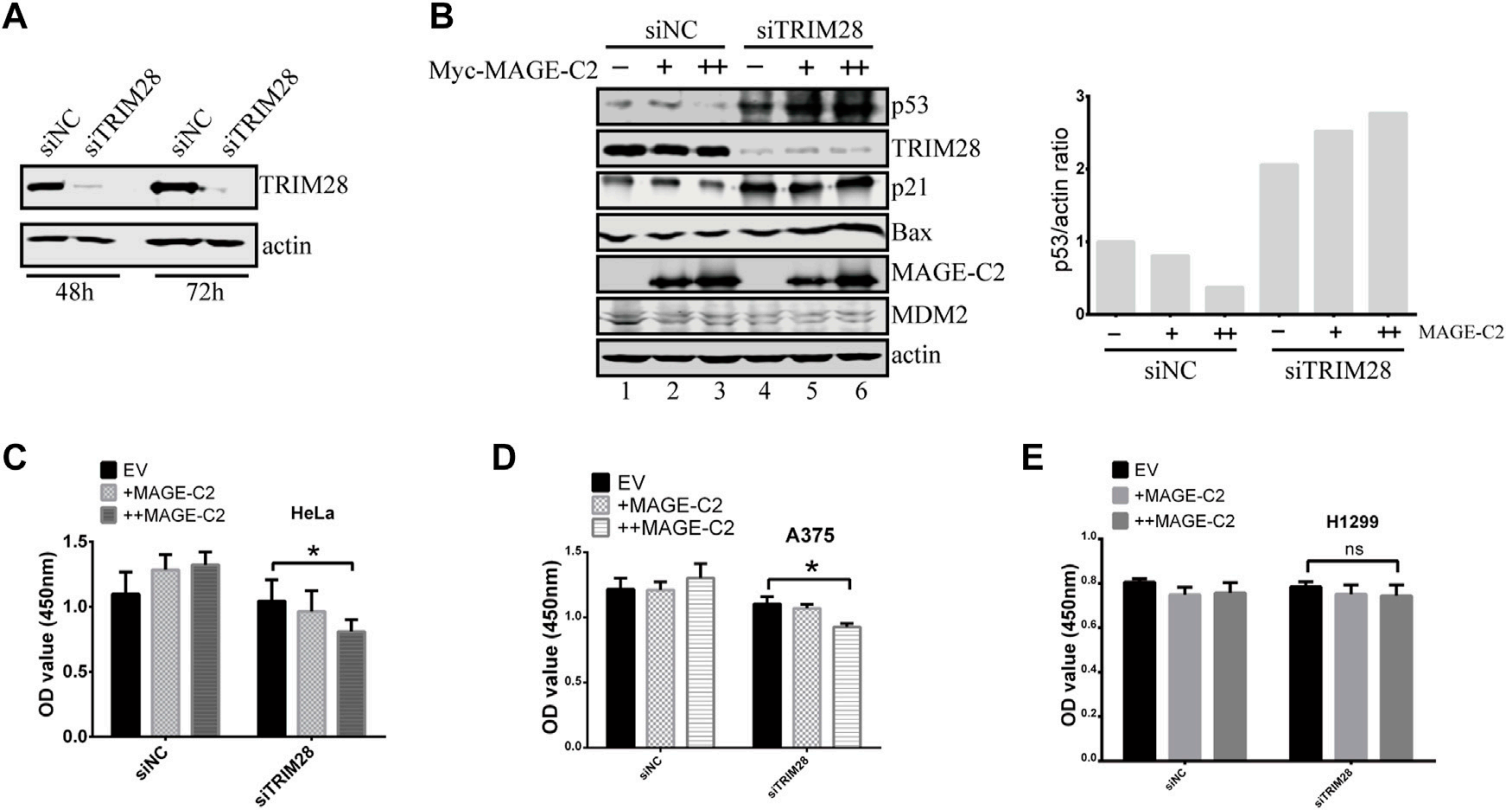
The inhibitory role of MAGE-C2 in p53 ubiquitination and cell proliferation is antagonized by TRIM28. **(A)** The RNA interference was utilized to knock down TRIM28. siRNA transfection was performed and the protein levels of TRIM28 were significantly decreased within 72 h. **(B)** The protein levels of p53 and other proteins were detected by using Western blot with indicated antibodies. HeLa cells were transfected with RNAi–TRIM28 and wild-type Myc-MAGE-C2 followed by immunoblotting with the indicated antibodies. **(C)** CCK-8 cell proliferation assay was performed to evaluate the cell viability in HeLa cells at 36 h. Average and SD are shown here. *p < 0.05. +: 1 μg MAGE-C2, ++: 2 μg MAGE-C2. **(D)** CCK-8 cell proliferation assay was performed to evaluate the cell viability in A375 cells at 36 h. Average and SD are shown here. *p < 0.05. +: 1 μg MAGE-C2, ++: 2 μg MAGE-C2. **(E)** CCK-8 cell proliferation assay was performed to evaluate the cell viability in H1299 cells at 36 h. Average and SD are shown here. ns denotes no significance. +: 1 μg MAGE-C2, ++: 2 μg MAGE-C2.

We then performed a series of experiments to examine if TRIM28 could antagonize the inhibitory role of MAGE-C2 in cells. To manipulate the protein expression of TRIM28, siRNA of TRIM28 was introduced into HeLa cells (Figure 3A). The knock down of TRIM28 resulted in the increase of p53 protein level (Figure 3B, lanes 1 and 4), and the gradient-expression of exogenous MAGE-C2 progressively reduced the p53 protein levels (Figure 3B, lanes 1–3) in control cells. However, in TRIM28-depleted cells, the expression of MAGE-C2 induced a further increased protein level of p53 (Figure 3B, lanes 4–6). To evaluate p53 activity, we also detected the protein level of two p53 transcriptional targets, p21 and BAX, and both proteins exhibited the similar expression pattern with p53 (Figure 3B). In summary, the expression of MAGE-C2 upregulated the level of p53 protein and subsequently upregulated the downstream target of p53 in the absence of TRIM28.

To confirm the antagonistic roles of TRIM28 on cell proliferation, we also performed the CCK-8 assay to examine the biological effect of TRIM28 and MAGE-C2. The overexpression of MAGE-C2 promoted the proliferation of HeLa cells (Figure 3C). However, the knockdown of TRIM28 and following expression of MAGE-C2 decreased the cell viability (Figure 3C), which were consistent with the protein patterns of p53 showed in Figure 3B. Similar results were obtained in melanoma cell line A375 cells which contain endogenous MAGE-C2 (Figure 3D). The same experiments were also carried out in p53-null lung cancer cell line H1299 (Wang et al., 2019), and there was almost no change in cell proliferation (Figure 3E). These results implied that the effect of MAGE-C2 and TRIM28 on cell proliferation is p53-dependent. Thus, our findings collectively indicated the differential roles of MAGE-C2 and TRIM28 that MAGE-C2 accumulates p53 protein level and reduces cell proliferation in the absence of TRIM28, while TRIM28 downregulates p53 protein level and accelerates cell proliferation.

We detected the transcriptional level of TRIM28 and MDM2 in HeLa cells using RT-qPCR, and found that the endogenous mRNA expression of TRIM28 was much higher than MDM2(Supplementary Figure S2G), that explained why we could not detect the increased proliferation in normal cells which TRIM28 may antagonize the inhibitory activity of MDM2 on p53 ubiquitination.

### TRIM28 Breaks the Interaction Between MAGE-C2 and Mouse Double Minute 2 to Promote p53 Ubiquitination

**FIGURE 4:**
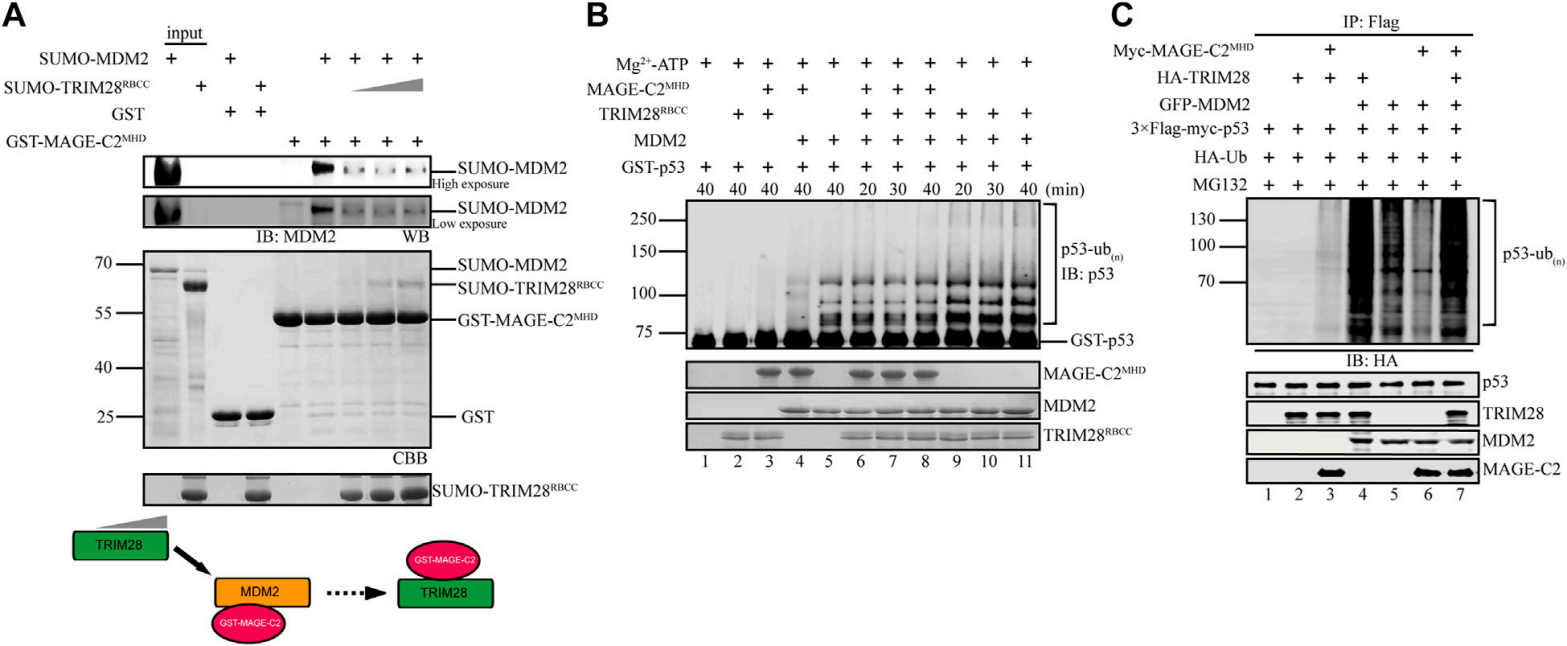
TRIM28 breaks the interaction between MAGE-C2 and MDM2 to promote p53 ubiquitination. **(A)** GST pulldown assay was used to validate the interaction relationship between TRIM28, MAGE-C2, and MDM2. MDM2 first bound to GST–MAGE-C2 on GST beads, and then TRIM28 was gradiently added. Beads with samples were immunoblotted with the MDM2 antibodies. **(B)** *In vitro* ubiquitination assay. His-ubiquitin, ubiquitin E1, UbcH5B E2, Mg-ATP, GST-p53, and the indicated MAGE-C2, MDM2, and TRIM28 proteins were incubated for specific time at 37°C and detected by using anti-p53 immunoblotting. **(C)** Ubiquitination assay in cells. HEK293T cells were transfected with HA-ubiquitin and the indicated plasmids. The level of p53 ubiquitination was determined by using anti-Flag M2-agarose redbeads purification and anti-HA Western blot.

MAGE-C2 directly binds to both TRIM28 and MDM2, suggesting a possible competition between TRIM28 and MDM2 for the binding to MAGE-C2. To distinguish their binding modes, we adopted a competition assay based on GST pulldown. As the result showed, MDM2 strongly bound to MAGE-C2 MHD domain on GST beads (Figure 4A, lane 6). However, the gradient addition of TRIM28 significantly decreased the binding level of MDM2 (Figure 4A). TRIM28 gradually replaced MDM2 and bound to MAGE-C2 MHD on GST beads in a dose-dependent manner. In contrast, MDM2 failed to squeeze TRIM28 out from MAGE-C2 MHD (Supplementary Figure S3). These results suggested that MAGE-C2 may hold higher binding ability to TRIM28 than MDM2.

To examine if TRIM28 could release the inhibitory role of MAGE-C2 on MDM2-dependent p53 ubiquitination, *in vitro* ubiquitination assays were performed in the presence or absence of TRIM28. To exclude the influence of TRIM28 E3 ligase activity, the assay only contained the unique E2 UbcH5B for MDM2, in which TRIM28 alone or together with MAGE-C2 did not exhibit E3 ligase activity (Figure 4B, lanes 2–3). Our results showed that MAGE-C2 alone dramatically inhibited the ubiquitination of p53 mediated by MDM2 (Figure 4B, lane 4), while TRIM28 alone certainly enhanced the p53 ubiquitination (Figure 4B, lanes 9–11). However, MAGE-C2 and TRIM28 together restored the p53 ubiquitination (Figure 4B, lanes 6–8) to the similar level as MDM2 alone did (Figure 4B, lane 5). Thus, our results suggested that TRIM28 releases the inhibitory role of MAGE-C2 on p53 ubiquitination by detaching MAGE-C2 from MDM2 *in vitro*.

We next carried out the cell-based ubiquitination assay utilizing HEK293T cells, in which TRIM28 and MDM2 could use their endogenous ubiquitin-conjugating enzyme (E2), respectively. In comparison to MDM2 alone, TRIM28 significantly enhanced the ubiquitination level of p53 mediated by MDM2 (Figure 4C, lanes 4–5), while the overexpression of MAGE-C2 remarkably inhibited the ubiquitination level of p53 (Figure 4C, lanes 5–6). Intriguingly, co-transfection with MDM2, TRIM28, and MAGE-C2 extremely increased the p53 ubiquitination level (Figure 4C, lane 7), suggesting that TRIM28 released the inhibition of MAGE-C2 on MDM2 and collaboratively promoted p53 ubiquitination. The extra ubiquitination level might be caused by the ligase activity of TRIM28 activated by MAGE-C2, although the E3 ligase activity of TRIM28–MAGE-C2 on p53 was weaker than MDM2 (Figure 4C, lane 3). Thus, these results suggested that TRIM28 competes with MDM2 for MAGE-C2 association, releases the inhibition of MAGE-C2 on MDM2, and results in amplifying p53 ubiquitination by both MDM2–TRIM28 and TRIM28–MAGE-C2.

### TRIM28 Collaborates With MAGE-C2 and Mouse Double Minute 2 to Promote Cell Proliferation and Migration

**FIGURE 5:**
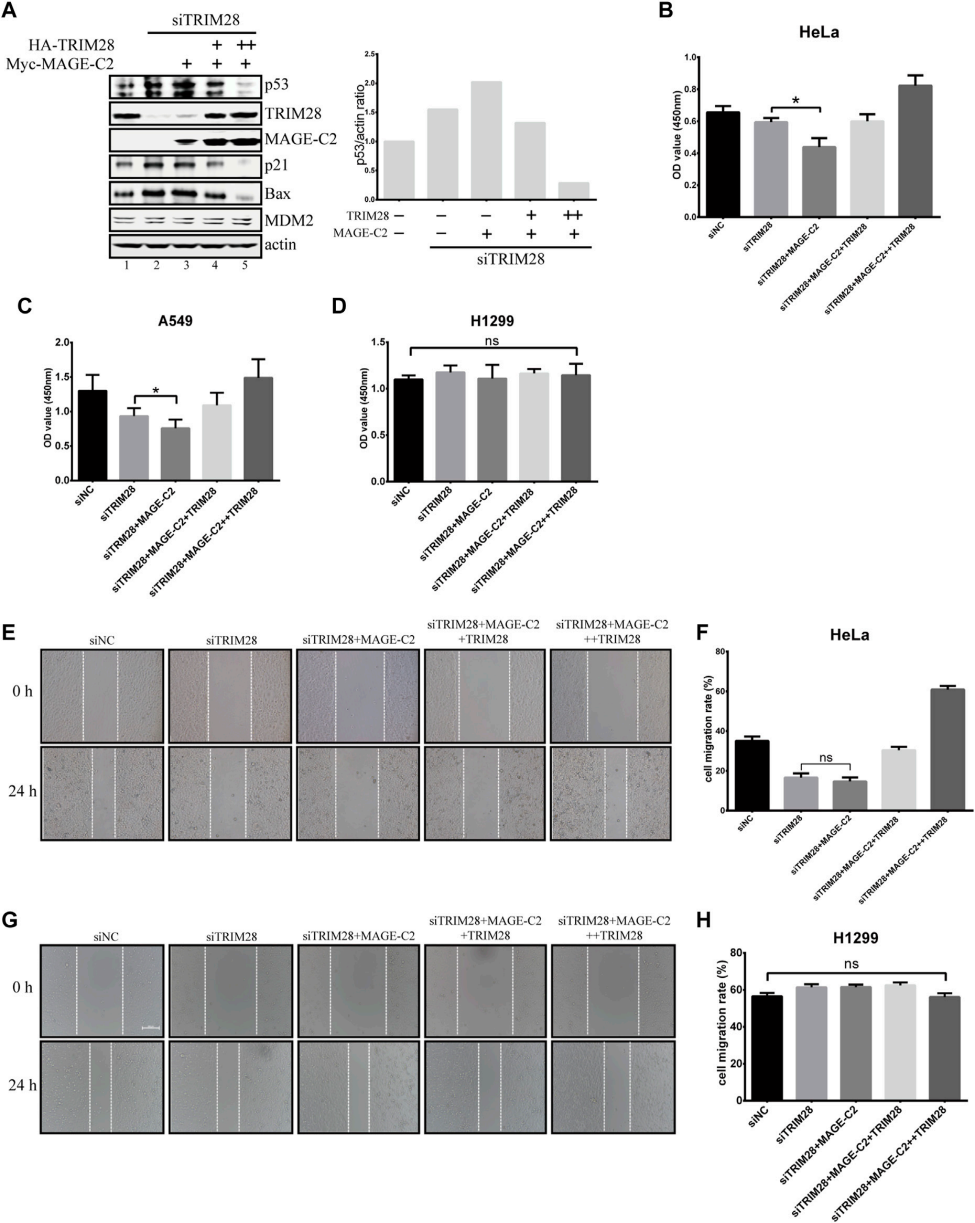
TRIM28 collaborates with MAGE-C2 and MDM2 to promote cell proliferation and migration. **(A)** TRIM28–RNAi was rescued by an siRNA-resistant HA-TRIM28. Cell lysates were collected 36 h after transfection and immunoblotted with the indicated antibodies. **(B**–**D)** CCK-8 assay was performed to evaluate the cell viability in HeLa cells **(B)**, A549 cells, **(C)** and H1299 cells **(D)**. Average and SD are shown here. *p < 0.05. ns denotes no significance. +: 0.5 μg TRIM28, ++: 1 μg TRIM28. **(E**,**F)** HeLa cells and **(G**,**H)** H1299 cells were observed and photographed with an inverted microscope. The reduction rate of wound within 24 h represents the migration rate of cells. Average and SD are shown here. ns denotes no significance. +: 0.5 μg TRIM28, ++: 1 μg TRIM28.

To further understand the biological function and relationship between TRIM28 and MAGE-C2, we constructed the siRNA-resistant TRIM28 plasmids to restore TRIM28 protein level in TRIM28-depleted HeLa cells. The depletion of endogenous TRIM28 increased the protein levels of p53, and the addition of ectopic MAGE-C2 further augmented the p53 protein levels (Figure 5A, lanes 1–3). Meantime, the CCK-8 assay and live/dead cell staining assay were performed to detect the proliferation effect. The depletion of TRIM28 slightly inhibited cell proliferation, and the exogenous MAGE-C2 exhibited further inhibition to cell proliferation (Figure 5B; Supplementary Figure S4A). These results suggested that MAGE-C2 could inhibit p53 degradation and cell proliferation in TRIM28-deficient cells. The expression of siRNA-resistant TRIM28 definitely restored TRIM28 protein to a comparable level however, the originally upregulated protein level of p53, as well as p21 and BAX, was substantially decreased (Figure 5A, lanes 4–5). Consistent with the change of p53 protein level, the exogenous MAGE-C2 together with siRNA-resistant TRIM28 rescued and accelerated cell viability (Figures 5B; Supplementary Figure S4A). Similar effect was also observed in another MAGE-C2–negative cells and lung cancer cell lines A549 (Figure 5C) but not in H1299 cells (Figures 5D; Supplementary Figure S4B). These data confirmed that TRIM28 collaborates with MAGE-C2 and MDM2 to promote cell proliferation in p53-dependent way in different types of cancer cells.

Due to the function of p53 in cell migration and invasion (Choi et al., 2020; Li and Zhang, 2020; Lv et al., 2020), the wound healing assays were also performed to examine the migration effect of TRIM28 and MAGE-C2 in HeLa cells. The depletion of TRIM28 significantly decreased the cell migration rate in comparison with the empty vector, and additional MAGE-C2 slightly exhibited further decline (Figures 5E,F). However, TRIM28 overexpression not only restored the migration of HeLa cells but also strongly promoted the cell motility (Figures 5E,F). As expected, H1299 cells showed no significant difference under the same treatment (Figures 5G,H).

Taken together, these results suggested that TRIM28 is indispensable for downregulating the p53 protein level and accelerating cell proliferation and migration through preventing the inhibitory effect of MAGE-C2 and stimulating the E3 activity of MDM2 on p53 simultaneously.

## Discussion

As an important tumor suppressor, the function and regulation mechanisms of p53 have gained much attention (Klibanov et al., 2001; Perwez Hussain and Harris, 2006; Pflaum et al., 2014). The E3 ligase MDM2 is a major regulator of p53 and cooperates with TRIM28 to stimulate p53 ubiquitination and subsequent degradation (Momand et al., 2000; Taranto et al., 2005; Wang et al., 2005; Levav-Cohen et al., 2014). Additionally, MAGE-C2 activates the ligase activity of TRIM28 to ubiquitinate p53 independently. However, the biochemical mechanisms and cellular function of MAGE-C2 and TRIM28 in MDM2-mediated p53 ubiquitination remain unclear. Here, our evidence provides a working model of how MAGE-C2 and TRIM28 regulate p53 protein level and cell proliferation through MDM2 (Supplementary Figure S5). MAGE-C2 directly interacts with MDM2 *via* its highly conserved MHD domain and inhibits the E3 ligase activity of MDM2 to retain the p53 protein levels *in vitro* and in TRIM28-deficient cells. More importantly, TRIM28 preferentially interacts with MAGE-C2 to compete against MDM2, which releases the inhibitory role of MAGE-C2 on MDM2-dependent p53 ubiquitination. TRIM28 restores and amplifies p53 ubiquitination by either acting as an E3 ligase activated by MAGE-C2 or stimulating MDM2. Our model clarifies the molecular relationship between two major E3 systems TRIM28–MDM2 and TRIM28–MAGE-C2 in p53 ubiquitination pathways. Our data also highlight the important function of TRIM28 as a key regulator to downregulate p53 protein level and eventually promote cell proliferation and migration. In summary, this study would be helpful for us to deeply understand the regulation mechanism of the tumor suppressor p53.

MAGE family members could bind to different E3 ring proteins through their conserved MHD domain, but the binding regions on E3 proteins may be variable in primary and second structures (Doyle et al., 2010). This property of MAGE family may lead to distinct functions with different partners. In our study, MAGE-C2 binds to both E3 ligases MDM2 and TRIM28 but with different outcomes, that MAGE-C2 inhibits E3 ligase activity of MDM2 while accelerates TRIM28. MAGE-A2 has also been shown to associate with MDM2 and function as an inhibitor of MDM2 but without any effect on p53 turnover in cell (Marcar et al., 2015), presumably because of the existence of TRIM28 in cell according to our conclusion.

Recently RLIM, also called RNF12, has been reported to act as an E3 ligase to promote MDM2 ubiquitination (Jin et al., 2021), and it could induce the degradation of MDM2, and finally regulate the cell proliferation through p53. The performances induced by both RLIM and MAGE-C2 could be overcome by TRIM28, but their molecular mechanisms are totally different. RLIM could induce the degradation of MDM2 and decrease the protein level of MDM2 while MAGE-C2 inhibits MDM2 E3 ligase activity. The protein level of MDM2 is basically stable in all our experiments. These two pathways may work together or separately to monitor p53-related outcomes.

Here, we provide an alternative mechanism of MAGE-C2, TRIM28, and MDM2 to regulate p53 protein level and finally affect cell proliferation and migration. Other members of MAGE family could also function in diverse mechanisms to regulate cell proliferation, which need to be further studied in the future.

## Data Availability

The original contributions presented in the study are included in the article/Supplementary Material; further inquiries can be directed to the corresponding author.
