# Supplementary figures and images for: The Dual Roles of MAGE-C2 in p53 Ubiquitination and Cell Proliferation Through E3 Ligases MDM2 and TRIM28
Source: Front Cell Dev Biol. 2022 Jul 19;10:922675. doi: 10.3389/fcell.2022.922675 (PMC9344466; doi:10.3389/fcell.2022.922675)

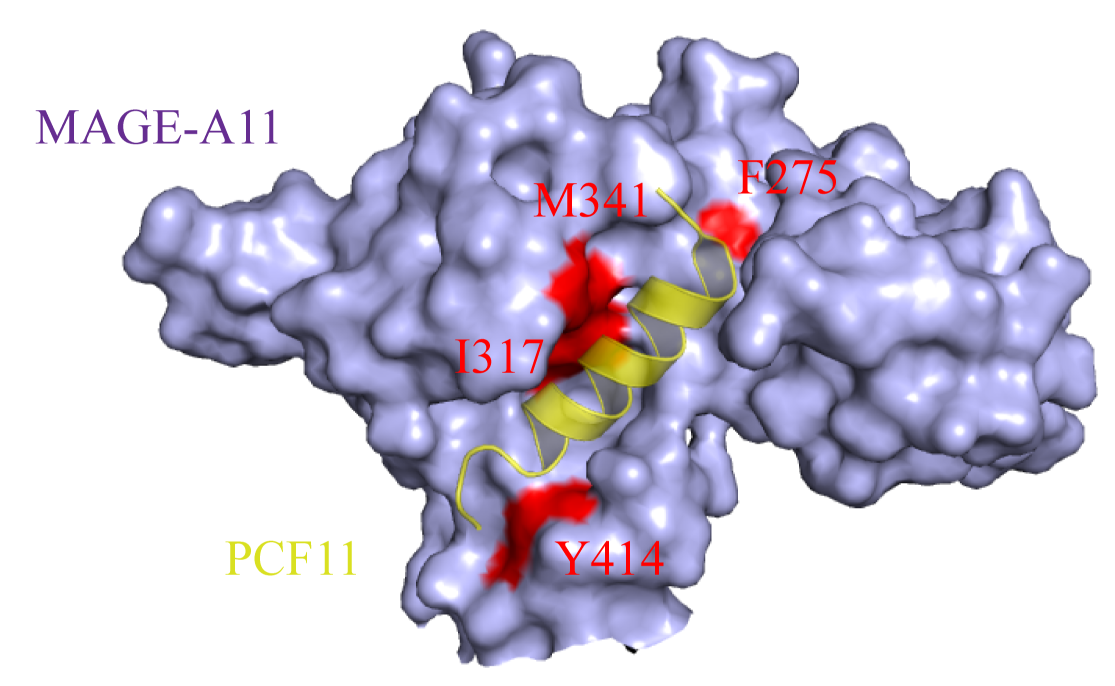

Supplement: Supplementary file 1 [file Image1.tif]
