# Supplementary material for: The Dual Roles of MAGE-C2 in p53 Ubiquitination and Cell Proliferation Through E3 Ligases MDM2 and TRIM28
Source: Front Cell Dev Biol. 2022 Jul 19;10:922675. doi: 10.3389/fcell.2022.922675 (PMC9344466; doi:10.3389/fcell.2022.922675)
Supplement: Supplementary file 2 [file DataSheet1.PDF]

Figure. S1 MAGE-C2 inhibits MDM2-mediated p53 ubiquitination through direct interaction

(A) *In vitro* ubiquitination assay. His-ubiquitin, ubiquitin E1, UbcH5B E2, Mg-ATP, GST-p53 and the MAGE-C2 truncation proteins, MDM2 proteins were incubated for the indicated time at 37°C and detected by anti-p53 immunoblotting.

(B) Crystal structure of MAGE-A11 complex with substrate PCF11 (PDB:6WJH). Site mutations (F193A, I232R, V256R, F329R) in the MHD domain of MAGE-C2 are corresponding to the SBC mutant of MAGE-A11.

Figure. S2 TRIM28 interacts with MAGE-C2 and promotes p53 ubiquitination

(A) Overexpression of MAGE-C2 induced p53 degradation. HeLa cells were transfected with wild-type Myc-MAGE-C2 followed by immunoblotting with the indicated antibodies 36 h later.

(B) CCK-8 cell proliferation assay was performed to evaluate the cell viability in HeLa cells at 36 h. Average and SD are shown here. $^*P < 0.05$. +: 1 μg MAGE-C2, ++: 2 μg MAGE-C2.

(C) Interactions between MAGE-C2 and TRIM28 were validated by GST pulldown assay. GST was included as an irrelevant negative control protein.

(D) and (F) Ubiquitination assays were carried out in HEK293T cells, indicated plasmids were transfected and ubiquitinated-p53 was detected by anti-p53 immunoblotting.

(E) *In vitro* ubiquitination assay. His-ubiquitin, ubiquitin E1, UbcH5B E2 (corresponding to MDM2), Mg-ATP, GST-p53 and the indicated MAGE-C2, MDM2, TRIM28 proteins were incubated for 40 min at 37°C and detected by anti-p53 immunoblotting.

(G) The mRNA expression of TRIM28 and MDM2 in HeLa cells, average and SD are shown here. $^{****}P < 0.0001$.

Figure. S3 MAGE-C2 hold higher binding ability to TRIM28 than MDM2

GST pulldown assay was used to validate the interaction relationship between TRIM28, MAGE-C2 and MDM2. TRIM28 firstly bound to GST-MAGE-C2 on GST beads, then MDM2 was added gradiently. Beads with samples were immunoblotted with the MDM2 antibodies.

Figure. S4 TRIM28 collaborates with MAGE-C2 and MDM2 to promote cell proliferation

(A) HeLa cells and (B) H1299 cells were used for live/dead cell staining assay, and cells were observed immediately under a fluorescence microscope.

Figure. S5 TRIM28 antagonizes the inhibitory role of MAGE-C2 and accumulates p53 ubiquitination to promote cell proliferation

The working model of how MAGE-C2 and TRIM28 regulate p53 protein level and cell proliferation through MDM2.

Figure. S1

A.

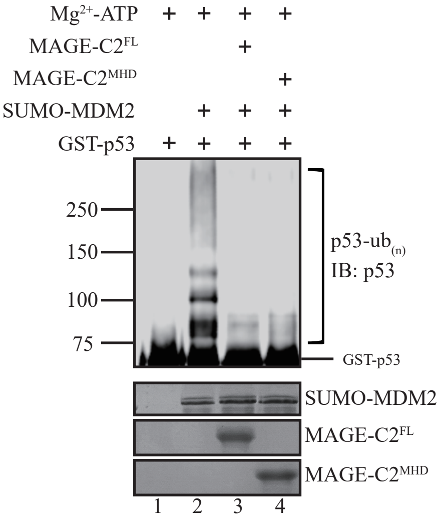

Mg2+-ATP
MAGE-C2FL
MAGE-C2MHD
SUMO-MDM2
GST-p53
250
150
100
75
p53-ub(n)
IB: p53
GST-p53
SUMO-MDM2
MAGE-C2FL
MAGE-C2MHD
1
2
3
4

B.

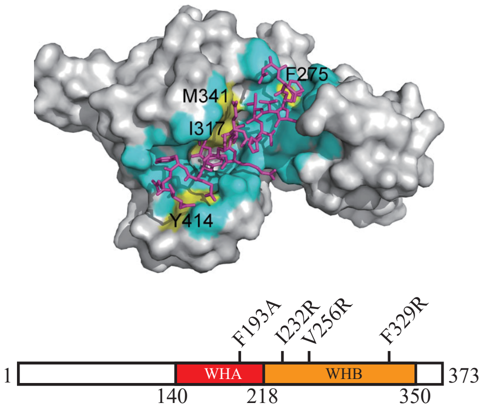

F275
M341
I317
Y414
F193A
I232R
V256R
F329R
1
WHA
WHB
373
140
218
350

Figure. S2

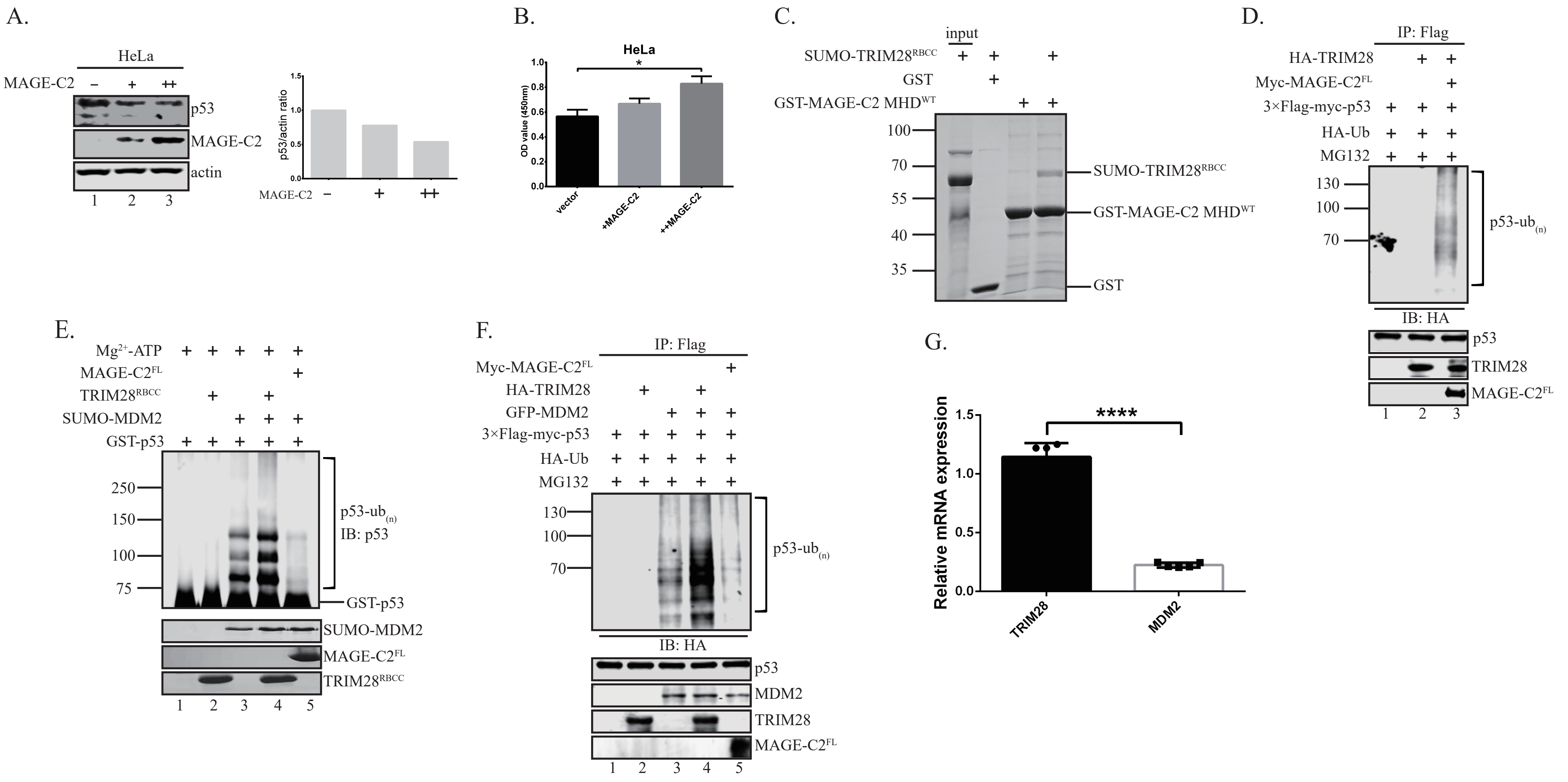
A.
HeLa
MAGE-C2 – + ++
p53
MAGE-C2
actin
1 2 3
p53/actin ratio
MAGE-C2 – + ++
B.
HeLa
*
OD value (450nm)
vector
+MAGE-C2
++MAGE-C2
C.
input
SUMO-TRIM28RBCC
GST
GST-MAGE-C2 MHDWT
100
70
55
40
35
SUMO-TRIM28RBCC
GST-MAGE-C2 MHDWT
GST
D.
IP: Flag
HA-TRIM28
Myc-MAGE-C2FL
3×Flag-myc-p53
HA-Ub
MG132
130
100
70
p53-ub(n)
IB: HA
p53
TRIM28
MAGE-C2FL
1 2 3
E.
Mg2+-ATP
MAGE-C2FL
TRIM28RBCC
SUMO-MDM2
GST-p53
250
150
100
75
p53-ub(n)
IB: p53
GST-p53
SUMO-MDM2
MAGE-C2FL
TRIM28RBCC
1 2 3 4 5
F.
IP: Flag
Myc-MAGE-C2FL
HA-TRIM28
GFP-MDM2
3×Flag-myc-p53
HA-Ub
MG132
130
100
70
p53-ub(n)
IB: HA
p53
MDM2
TRIM28
MAGE-C2FL
1 2 3 4 5
G.
****
Relative mRNA expression
1.5
1.0
0.5
0.0
TRIM28
MDM2

Figure. S3

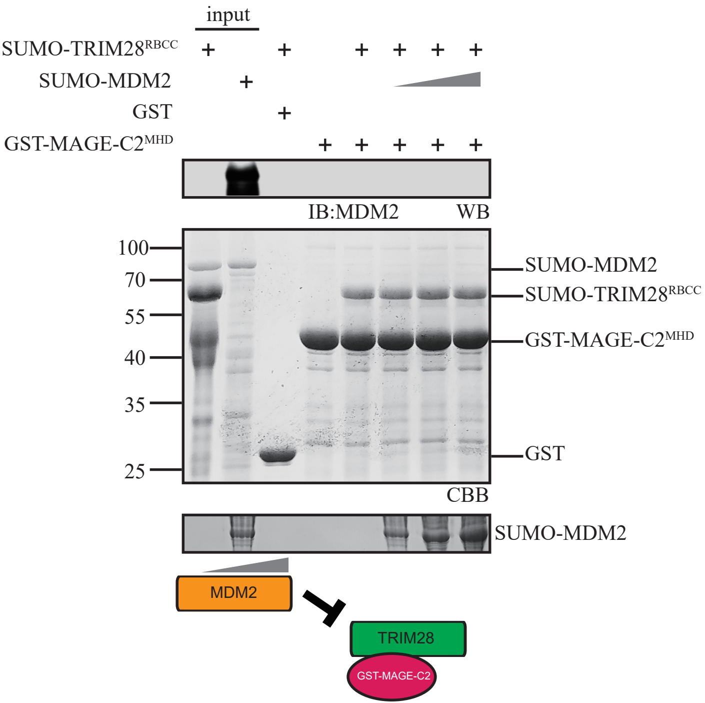
input
SUMO-TRIM28RBCC
SUMO-MDM2
GST
GST-MAGE-C2MHD
IB:MDM2
WB
100
70
55
40
35
25
SUMO-MDM2
SUMO-TRIM28RBCC
GST-MAGE-C2MHD
GST
CBB
SUMO-MDM2
MDM2
TRIM28
GST-MAGE-C2

Figure. S4

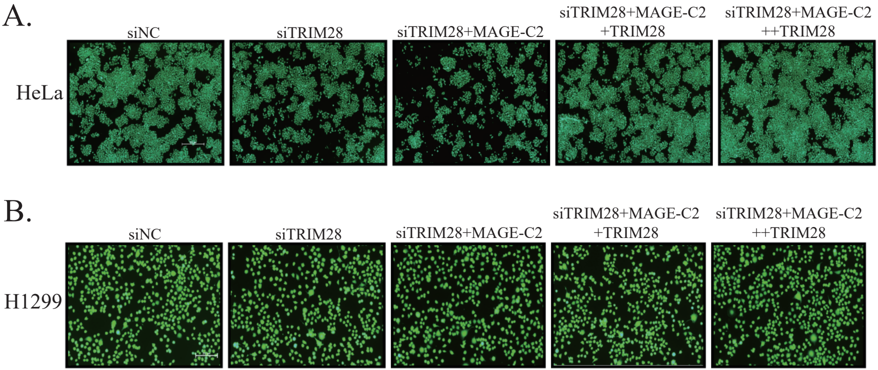
A.
siNC
siTRIM28
siTRIM28+MAGE-C2
siTRIM28+MAGE-C2
+TRIM28
siTRIM28+MAGE-C2
++TRIM28
HeLa
B.
siNC
siTRIM28
siTRIM28+MAGE-C2
siTRIM28+MAGE-C2
+TRIM28
siTRIM28+MAGE-C2
++TRIM28
H1299

Figure. S5

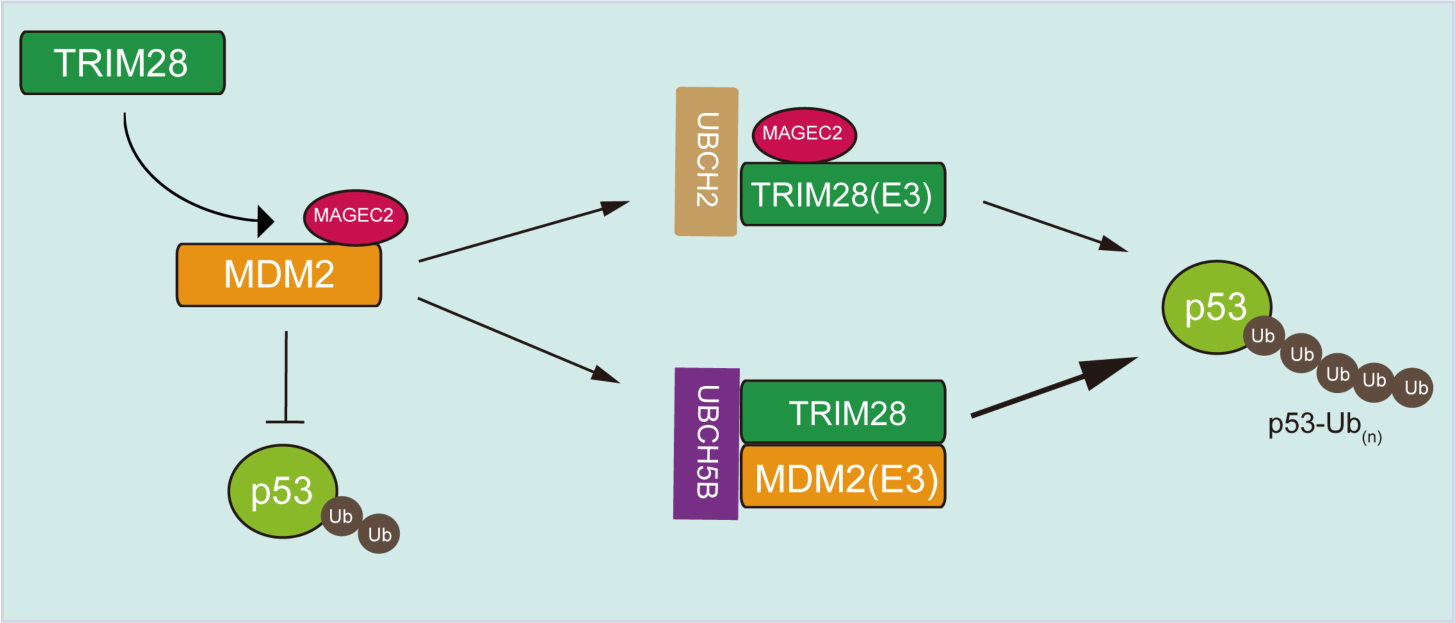

TRIM28
MAGEC2
MDM2
p53
Ub
Ub
UBCH2
MAGEC2
TRIM28(E3)
UBCH5B
TRIM28
MDM2(E3)
p53
Ub
Ub
Ub
Ub
Ub
p53-Ub(n)
